# The Administrative Side of Sanatorium Work

**Published:** 1909-08-21

**Authors:** 


### Nursing Administration.

### THE ADMINISTRATIVE SIDE OF SANATORIUM WORK.

### IV.—THE WORKING STAFF.

It is always a difficult matter to use miscellaneous labour to good advantage. The attempts of charitable and municipal societies to make work even for men in the prime of life and health have usually been lamentable failures from the financial point of view. When the labour available is that of consumptives, forced to work under special conditions, and to lead an invalid life during the process, the difficulties are increased tenfold. There is no doubt that the influence of a practical and zealous matron is one of the principal factors in making working colonies a success. The first essential to contentment is the home-like feeling which a woman can impart without thinking about it, and when this is once established the patients are eager to remain, put their best work into their allotted duties, and keep the machine oiled with a good will.

The staff needed to organise the male patients' work can be mainly recruited from among the patients themselves. A foreman, who shall be responsible to the matron or superintendent for the hours during which each patient is employed, is the most important official. If he has himself gone through the stages of recovery in this or some other sanatorium, he will be better suited to overlook similar labours on the part of the patients. He will be instructed to keep his eyes open for any signs of flagging among new recruits, and will be able to judge what demands are being made on the strength. Moreover, and this is an important factor in the situation, he will be far cheaper than outside helpers. A suitable foreman ought to be secured for about 5s. a week, with the proviso that he resides in the colony, gets full board, and is admitted to the advantages of "living the life."

Out of doors there must be a head gardener and probably a boy under him. Plenty of labour will be available among the patients for keeping the place in spick-span order; but unless the gardener is well trained, much of the genuine enthusiasm which townsmen always exhibit in the culture of flowers and vegetables will run to waste. We fear that in this department false economy often plays a detrimental part, and that the colony, with merely a working man as gardener, gets in consequence little profit out of the opportunities offered by extensive grounds and superabundant labour. The ordinary gardener is in truth seldom fitted for the supervision of an estate. There should be an efficient foreman gardener living in a cottage or lodge attached to the colony, and experienced in the art of developing all the possibilities offered by the available space. When it is remembered that a labouring gardener gets £52 a year for the very moderate services he renders, it will be seen that it would be enormously to the advantage of the colony to pay £80 or £90 with house and get a superior man. It is extremely improbable that the chances of institution life would bring the right kind of patient to undertake this department, on which much of the onus of making the establishment pay will rest.

It may often happen that more labour is available than can profitably be employed in workshops and garden, and the addition of a small farm for supplying produce to the inmates can in favourable localities be made to pay its way fairly well. Dairy farming is unsuitable for obvious reasons, but these reasons do not hold good with poultry keeping, and a well-conducted poultry farm on modern lines should prove an attractive and paying concern. Very little capital is required to start such an industry in a colony able to supply the labour for building fowl-houses and laying out runs. It is capable of gradual expansion, as occasion offers, and can absorb, under competent superintendence, a large amount of unskilled labour.

As the colony progresses, a variety of old patients will be added to the staff, it being considered part of the functions of the sanatorium to provide work and a home for such men as can hardly live under ordinary conditions, and are likely to be useful. For about 5s. a week and often for less, the services of good carpenters, painters, and other artisans can be secured for the colony. In addition to wages, such working patients ought not to cost more than from 8s. to 10s. a head for board, washing, and minor expenses, without reckoning establishment charges.

The employment of female patients to profitable purpose presents more difficulties. At Frimley, the women help in the garden, the laundry and the outdoor work generally, but in small establishments it is often found that those whose circumstances admit of their leaving their own homes for a long course of treatment are not such as are accustomed to hard manual labour, and they do not take very kindly to it. To confine them to such sedentary occupations as lace-making, is a doubtful benefit since half the battle is restoration to active habits of life. A judicious blend of needlework and spells of labour in the garden and farm is perhaps the best solution of the difficulty so far arrived at.

It is true of this as of all institution work that one of the chief secrets of economy lies with the housekeeping. It is less necessary than in hospitals to separate the board of inmates from that of the staff in the reckoning, for in truth right through the household the inmates are being trained to perform the duties of the staff, and their diet knows little distinction. The cost of boarding the household, including patients, at Frimley, is 6s. 6½d. a head a week, and this may well be taken as a model, both as regards cost and quality. In conclusion it must be always kept in view that to run a sanatorium at the minimum cost it is essential that the permanent staff be kept small in numbers, and that every member of it be highly trained for the work, and well paid. This is the only road to economy.

